# Oxidative stress and inflammation distinctly drive molecular mechanisms of diastolic dysfunction and remodeling in female and male heart failure with preserved ejection fraction rats

**DOI:** 10.3389/fcvm.2023.1157398

**Published:** 2023-06-08

**Authors:** Saltanat Zhazykbayeva, Roua Hassoun, Melissa Herwig, Heidi Budde, Árpád Kovács, Hans Georg Mannherz, Ibrahim El-Battrawy, Attila Tóth, Wolfgang E. Schmidt, Andreas Mügge, Nazha Hamdani

**Affiliations:** ^1^ Department of Cellular and Translational Physiology, Institute of Physiology, Ruhr University Bochum, Bochum, Germany; ^2^ Institut für Forschung und Lehre (IFL), Molecular and Experimental Cardiology, Ruhr University Bochum, Bochum, Germany; ^3^ Department of Cardiology, St. Josef-Hospital, UK RUB, Ruhr University Bochum, Bochum, Germany; ^4^ Department of Anatomy and Molecular Embryology, Ruhr University Bochum, Bochum, Germany; ^5^ Department of Cardiology and Angiology, Bergmannsheil University Hospitals, UK RUB, Ruhr University of Bochum, Bochum, Germany; ^6^ Division of Clinical Physiology, Department of Cardiology, Faculty of Medicine, University of Debrecen, Debrecen, Hungary; ^7^ Research Centre for Molecular Medicine, University of Debrecen, Debrecen, Hungary; ^8^ Department of Medicine I, St. Josef Hospital, UK RUB, Ruhr-University Bochum, Bochum, Germany

**Keywords:** diastolic dysfunction, sex differences, mechanisms, oxidative stress, inflammation

## Abstract

Heart failure with preserved ejection fraction (HFpEF) is a complex cardiovascular insufficiency syndrome presenting with an ejection fraction (EF) of greater than 50% along with different proinflammatory and metabolic co-morbidities. Despite previous work provided key insights into our understanding of HFpEF, effective treatments are still limited. In the current study we attempted to unravel the molecular basis of sex-dependent differences in HFpEF pathology. We analyzed left ventricular samples from 1-year-old female and male transgenic (TG) rats homozygous for the rat Ren-2 renin gene (mRen2) characterized with hypertension and diastolic dysfunction and compared it to age-matched female and male wild type rats (WT) served as control. Cardiomyocytes from female and male TG rats exhibited an elevated titin-based stiffness (F_passive_), which was corrected to control level upon treatment with reduced glutathione indicating titin oxidation. This was accompanied with high levels of oxidative stress in TG rats with more prominent effects in female group. In vitro supplementation with heat shock proteins (HSPs) reversed the elevated F_passive_ indicating restoration of their cytoprotective function. Furthermore, the TG group exhibited high levels of proinflammatory cytokines with significant alterations in apoptotic and autophagy pathways in both sexes. Distinct alterations in the expression of several proteins between both sexes suggest their differential impact on disease development and necessitate distinct treatment options. Hence, our data suggested that oxidative stress and inflammation distinctly drive diastolic dysfunction and remodeling in female and male rats with HFpEF and that the sex-dependent mechanisms contribute to HF pathology.

## Introduction

Worldwide the female sex is more affected after menopause by cardiovascular diseases with high morbidity and mortality rates. However, little is known about sex-dependent differences in mechanisms that drive disease prognosis and therapy outcomes (1). Considering the increasing cardiovascular morbidity and mortality in both sexes and the growing evidence of sex differences in cardiovascular diseases (2), the therapeutic advances in heart failure (HF) apply essentially exclusively to men and have not been investigated sufficiently in women even in breakthrough clinical trials women are underrepresented (3–5). Furthermore, female patients with heart failure with preserved ejection fraction (HFpEF) showed evidence of greater diastolic dysfunction associated with higher left ventricular (LV) filling pressure and diastolic stiffness as compared to male HFpEF patients (6).

Oxidative stress and inflammation are ascribed a central role in HFpEF pathophysiology. Both mechanisms mediate diastolic dysfunction via endothelial, extracellular matrix (ECM), and cardiomyocyte dysfunction (7). Despite the well-established contribution of redox imbalance to cardiomyocyte dysfunction, studies in both sexes are contradictory and with difference in functioning outcomes. For instance, studies on 9-week-old Wistar rats (castrated or sham-operated) have shown that oxidative stress was higher in male than in female rats (8), perhaps due to a lower induction of vascular reactive oxygen species (ROS) levels in female rats (9). Indeed, higher levels of oxidative stress biomarkers were detected in young male compared to female rats of the same age (9). In addition, recent evidence indicated that there are differences between men and women in the expression and activity of antioxidant enzymes (10), although a unified consensus is not yet apparent (11). This implies differences in the speed of the shift between oxidants and antioxidants, but does not explain the differences in oxidants between women and men with HF. Therefore, the question remains whether there are differences between both sexes at the molecular level caused by distinct oxidative stress levels. This would be important for the development of new treatment options with better efficiency for both sexes.

In addition to oxidative stress, inflammation contributes to HF development and progression linking excessive ROS with cytokine formation that results in downstream signaling pathways (12, 13). Like for oxidative stress, it remains unclear whether inflammatory events lead to sex different responses. The female sex is associated with higher susceptibility to inflammatory events and autoimmune diseases (14). On the other hand, women are less affected by inflammation than men, possibly through the protection provided by estrogens (11). We have previously provided evidence on the detrimental effects of redox imbalance on cardiomyocyte function (15, 16). The mechanisms by which oxidative damage occurs include ROS-mediated oxidative modification of myofilament proteins and/or indirect modulation of signaling pathways leading to the accumulation of oxidized proteins (17). Under various stress conditions, cardiomyocyte function is maintained via protein quality control system (PQS), which mediate the correction of misfolded proteins and/or the clearance of aberrant proteins that cannot be rescued (18). However, ROS might induce several impairments in PQS components leading to the accumulation of protein aggregates and cardiomyocyte dysfunction (19, 20). In experimental models an enhanced cardiac protection was demonstrated in female rats compared with males after trauma-induced hemorrhage, which was associated with estrogen-promoted upregulation of myocardial-specific heat shock proteins (HSPs) (21) indicating sex-based differences in PQS. In the current study, we aimed to investigate the sex-dependent differences in molecular pathways that contribute to HF pathology, especially the diastolic compliance in response to hypertensive conditions in male vs. female mRen2 transgenic (TG) rats.

## Methods

### Animal model

All animal care and experimental procedures were approved by the Ethical Committee of the University of Debrecen (Ethical Statement No. 1/2013/DE MÁB) in accordance with the Directive 2010/63/EU of the European Parliament. Female (*n* = 8) and male (*n* = 13) homozygous transgenic (TG) rats carrying the mouse Ren-2 renin gene (mRen2) at 1 year of age were compared with age-matched female (*n* = 12) and male (*n* = 6) non-transgenic wild type (WT) control rats from our in-bred colonies (22). Parent female and male animals were originally obtained from the Max Delbrück Center for Molecular Medicine in the Helmholtz Association (MDC), Berlin-Buch, Germany. No medication (e.g., antihypertensive drug) was administered to study subjects, and animals were fed a standard chow and tap water *ad libitum*. At 1 year of age, animals were sacrificed, hearts and left ventricles (LV) were quickly excised and weighed, and then further dissected in isolating solution (1.0 mM MgCl_2_, 100.0 mM KCl, 2.0 mM EGTA, 4.0 mM ATP, and 10.0 mM imidazole, pH 7.0; all chemicals from Sigma-Aldrich, St. Louis, MO), snap frozen in liquid nitrogen, and stored at 80°C until further use.

### Echocardiography

Transthoracic echocardiography with a General Electric Vivid E9 ultrasound system equipped with a linear 14.1 MHz i13l probe (General Electric, Fairfield, CT, USA) was performed on 1 year old rats under light in cardiomyocytesb y combination of ketamine and xylazine (50 mg/kg and 5 mg/kg body weight, respectively). Parasternal long axis M-mode was obtained at the level of the papillary muscles for the morphology of left ventricular (LV) such as wall thickness and internal diameter and also to assess systolic function such as ejection fraction (EF). Early diastolic filling peak velocity (E), late filling peak velocity (A), E-wave deceleration time (DT) and isovolumetric relaxation time (IVRT) were recorded by pulsed-wave Doppler. All Images were analyzed off-line by EchoPAC clinical workstation software (General Electric).

### Quantification of tissue oxidative stress

Total glutathione (GSH) in myocardial homogenates (*n* = 6 LV sample/group) was determined in triplicate with a colorimetric glutathione assay kit (CS0260, Sigma-Aldrich) according to manufacturer's instructions and as previously described (23).

### Western blot analysis

LV tissue samples were solubilized in a modified Laemmli buffer (50 mM Tris–HCl at pH 6.8, 8 M urea, 2 M thiourea, 3% SDS w/v, 0.03% ServaBlue w/v, 10% v/v glycerol, 75 mM DTT, all from Sigma-Aldrich, St. Louis, MO, USA), heated for 3 min at 96°C and centrifuged for 3 min at 4°C and 14,000 rpm. From LV supernatant, 20 *μ*g protein/lane was loaded and separated by electrophoresis using 12% or 15% SDS gels, which were run at 90 V for 20 min followed by 125 V for 90 min. After SDS-PAGE, the gels were blotted onto polyvinylidene difluoride (PVDF) membranes (Immobilon-P 0.45 *μ*m; Merck Millipore, Burlington, MA, USA). Blots were blocked with 5% bovine serum albumin (BSA) in Tris-buffered saline with Tween (TBST) for 1 h at room temperature (RT) and subsequently incubated with primary antibodies overnight at 4°C (Table 1). We used GAPDH (Sigma, 1:10,000) for comparison of protein load. After washing with TBST, primary antibodies were detected with HRP-conjugated secondary anti-rabbit or anti-mouse antibodies (1:10,000) and enhanced chemiluminescence (Clarity Western ECL Substrate, BioRad, Munich, Germany). Imaging was carried out with a ChemiDoc Imaging system (BioRad). Stained protein bands were quantified by densitometry using the Image Lab software (version 6.1., Bio-Rad, Hercules, CA, USA) and Multi Gauge V3.2 software. Finally, the signals obtained for the amounts of total protein and phosphorylated protein were normalized to signals obtained from GAPDH stains referring to the entire protein amount transferred. Phosphoproteins are shown as ratio of total protein. The obtained density values are expressed in arbitrary units (a.u).

**Table 1:** Primary antibody list.

Antibody	Catalogue number	Company	Dilution
Alpha—B-crystallin	Ab13497	Abcam	1:1,000
Cathepsin L	Sc-32320	Santa Cruz Biotechnology	1:1,000
Calpain 1 Large Subunit (Mu-type)	2556S	Cell Signaling	1:1,000
Caspase 1	2225S	Cell Signaling	1:1,000
Caspase 3	14220S	Cell Signaling	1:1,000
Caspase 9 p10	Sc-7885	Santa Cruz Biotechnology	1:1,000
HSP 27 (rodent preferred)	2442S	Cell Signaling	1:1,000
HSP 70	Ab2787	Abcam	1:1,000
IL 6	P620	Invitrogen	1:1,000
IL 18	PA5-80719	Invitrogen	1:1,000
LC 3 A/B	12741S	Cell Signaling	1:1,000
Total mTor	2983S	Cell Signaling	1:1,000
Phospho—mTor (S2448)	2971S	Cell Signaling	1:1,000
Total NF-*κ*appaB p65	8242S	Cell Signaling	1:1,000
Phospho—NF-κappaB p65 (S536)	3033S	Cell Signaling	1:1,000
Nox2	MA5-35348	Invitrogen	1:1,000
Nox4	MA5-32090	Invitrogen	1:1,000
SQSTM1/P62	39749S	Cell Signaling	1:1,000
TnF alpha	AMC3012	Invitrogen	1:1,000
GAPDH	G9545-200UL	Sigma	1:10,000

### Titin expression and phosphorylation

To detect titin expression and phosphorylation, LV samples were solubilized in the modified Laemmli (buffer composition given above). Samples were heated at 96°C for 3 min, centrifuged for 3 min at 4°C at 14,000 rpm, and then separated by agarose strengthened 2% SDS-PAGE (24, 25). Gels were run at 2–4 mA constant current per gel for 16 h. Thereafter, western blotting was performed to measure the expression and total phosphorylation of titin. Following SDS-PAGE, proteins were blotted onto polyvinylidene difluoride (PVDF) membranes (Immobilon-P 0.45 *μ*m; Merck Millipore, Burlington, MA, USA). Blots were preincubated with 5% bovine serum albumin in Tris-buffered saline with Tween (TBST; containing: 10 mM Tris–HCl; pH 7.6; 75 mM NaCl; 0.1% Tween; all from Sigma-Aldrich) for 1 h at RT followed by primary antibody incubation overnight at 4°C. Titin phosphorylation was determined by an anti–phospho-serine/threonine antibody (ECM Biosciences LLC; PP2551; 1:500); for titin oxidation an anti-GSH antibody (ab19534, Abcam, 1:500) and for titin ubiquitination an anti –ubiquitin antibody (43124S, Cell signaling, 1:750) was used. Titin phosphorylation, oxidation and ubiquitination were visualized by HRP-conjugated secondary anti-rabbit or anti-mouse antibodies (1:10,000), which were used next day for 1 h at RT, then blots were treated with ECL (Clarity Western ECL Substrate, BioRad) for developing chemiluminescence signal. Chemiluminescence signals were normalized to signals obtained from Coomassie-stained PVDF membranes referring to the entire protein amount transferred. The results were quantitated by densitometry using Multi Gauge V3.2 software.

### Force measurements on isolated cardiomyocytes

Force measurements were performed on single de-membranated cardiomyocytes (*n* = 26–30/5–6 heart/group) as described before (26).

Briefly, LV samples were de-frozen in relaxing solution (containing in mM: 1.0 free Mg2+; 100 KCl; 2.0 EGTA; 4.0 Mg-ATP; 10 imidazole; pH 7.0), mechanically disrupted and incubated for 5 min in relaxing solution supplemented with 0.5% Triton X-100 (all from Sigma-Aldrich). The cell suspension was washed 5 times in relaxing solution. Single cardiomyocytes were selected under an inverted microscope (Zeiss Axiovert 135, 40x objective; Carl Zeiss AG Corp, Oberkochen, Germany) and attached with silicone adhesive between a force transducer and a high-speed length controller (piezoelectric motor) as part of a “Permeabilized Myocyte Test System” (1600A; with force transducer 403A; Aurora Scientific, Aurora, Ontario, Canada).

Cardiomyocyte Ca^2+^-independent passive force (F_passive_) was measured in relaxing buffer at room temperature within a sarcomere length (SL) range between 1.8 and 2.4 *μ*m. Force values were normalized to myocyte cross-sectional area calculated from the diameter of the cells, assuming a circular shape. F_passive_ was thereafter measured within a SL range between 1.8 and 2.4 *μ*m as described above.

The forces were recorded at baseline and after incubation with the antioxidant, reduced glutathione (GSH) 30 min (10 mM; Sigma-Aldrich) and/or recombinant human *αβ*-crystallin or HSP27 or HSP70 concentrations 1 mg/ml and caspase 3 inhibitor concentration 0,5 mg/ml. All incubations were performed for 20 min to 30 min in relaxing solution.

### Statistical analysis

Data are given as the mean values ±SEM. For statistical analysis of the two groups of parametric data Student's *t*-test was used, for non-parametric data Mann–Whitney test was used. For analysis of parametric data comparing more than two groups, 2-way ANOVA followed by Tukey's multiple comparisons test was used. *P* values were corrected for multiple comparisons by the Tukey method. For analysis of proportions, Fisher's exact test was used. The analysis was performed using GraphPad Prism 8. *P* values are two-sided and considered statistically significant if *P* < 0.05.

## Results

TG male group showed cardiac enlargement and LV hypertrophy appreciated from weight/TL and LV weight/TL ratios, which were significantly higher in TG males (46.84 ± 2.29) than those in either WT males (34.55 ± 1.96) or TG females (29.90 ± 2.63). Nonetheless, pulmonary congestion with the apparent dominancy of males could not be confirmed in TG animals because lung wet/dry weight ratios were unchanged. In addition liver wet/dry weight ratios were similar as well.

Additional data based on echochardiography analysis (ECG) showed for the TG male rats left ventricle diastolic dysfunction and in female TG rats a preserved but in male TG rats a reduced left ventricle ejection fraction. The male TG showed also left ventricle hypertrophy with the absence of LV dilation. Finally, TG animals showed impaired relaxation as evident from the mitral inflow pattern with a prolonged isovolumic relaxation time (IVRT), a prolonged deceleration time (DT), and decreased E/A ratio in both sexes.

### Sex and oxidative stress dependent alterations in titin-based cardiomyocyte stiffness

**Figure 1:**
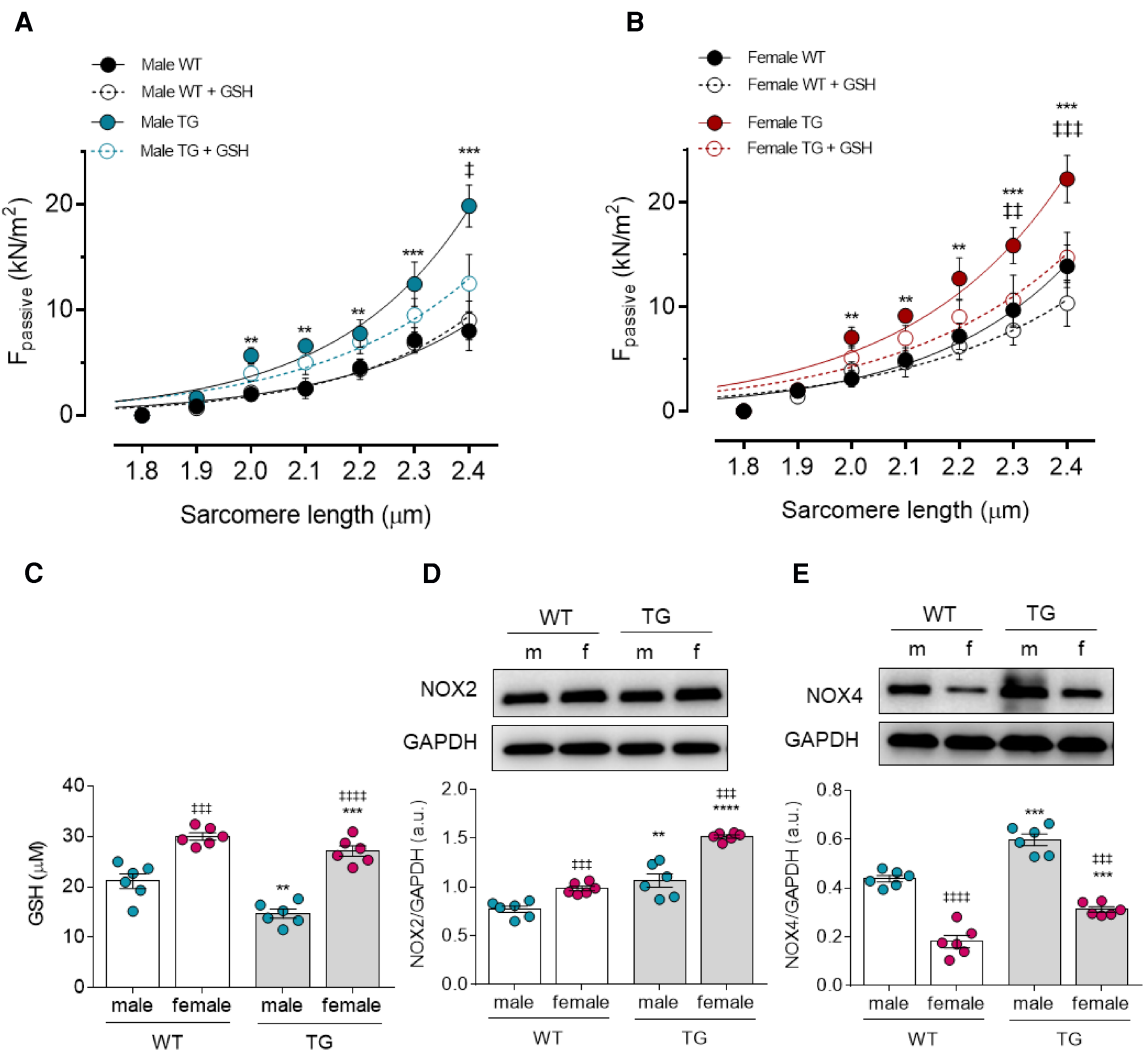
Cardiomyocyte passive stiffness (F**_pas_**_sive_) and oxidative stress parameters in male and female mRen2 and WTrats. F_passive_ before (baseline) and after *in vitro* reduced glutathione (GSH) treatment at sarcomere length 1.8–2.4 *μ*m in (**A**) male and (**B**) female TG and WT rats. (**C,D**) Comparison of passive stiffness lowering effect (*Δ*Fpassive) of GSH in TG females vs. TG males is shown at sarcomere length of 2.2 µm. (**E**) GSH concentration level, (**F**) Nicotinamideadenine-dinucleotide phosphate oxidase (NOX) 2 expression level and (**G**) NOX4 expression level. Data are shown as mean ± SEM; panels (**A,B**), (*n* = 26–30/5–6 heart/group): Data are shown as mean ± SEM; *n* = 6. Panel (**A,B**): *P < 0.05/**P < 0.01/***P < 0.001/****P < 0.001 female WT vs. female TG and male WT vs. male TG; ‡*P* < 0.05/‡‡*P* < 0.01/‡‡‡*P* < 0.001/‡‡‡‡*P* < 0.001 female TG vs. female TG + GSH and male TG vs. male TG + GSH. Panel (**C,D**): *P < 0.05/**P < 0.01/***P < 0.001/****P < 0.001 female WT vs. female TG and male WT vs. male TG; ‡*P* < 0.05/‡‡*P* < 0.01/‡‡‡*P* < 0.001/‡‡‡‡*P* < 0.001 female WT vs. male WT and female TG vs. male TG; ##*P* < 0.01/###*P* < 0.001/the difference of Fpassive after GSH beween female TG and male TG after GSH by 2-way ANOVA followed by Tukey's multiple comparisons test.

To investigate the effect of sex on diastolic dysfunction observed in TG animals, we measured F_passive_ in single-skinned cardiomyocytes at sarcomere lengths (SL) between 1.8 and 2.4 *μ*m. The cardiomyocytes were obtained from male and female rats and from healthy (WT) and transgenic (TG) rats before and after the treatment with reduced glutathione (GSH). Male TG cardiomyocytes showed significant increase in F_passive_ at SL 2.0 µm and above compared to male WT group. GSH treatment decreased the elevated F_passive_ in male TG cardiomyocytes, however, the reduction in F_passive_ was only significant at SL of 2.4 µm (Figure 1A). Similarly, F_passive_ was significantly increased in female TG cardiomyocytes at SL 2.0 and beyond compared with female WT group and could be significantly corrected at SL of 2.3 and 2.4 after GSH treatment (Figure 1B). F_passive_ of control cardiomyocytes from WT male as well as WT female rats remained unaltered in response to GSH treatment (Figures 1A,B). The direct comparison between female vs. male TG and calculating the difference before and after GSH (*Δ*Fpassive) female vs. male TG showed the great benefit of female TG from GSH compared to male TG (Figures 1C,D).

Since GSH treatment was effective in reducing F_passive_, we examined GSH expression level (Figure 1E). Interestingly, both female and male TG rats showed reduced GSH level in comparison with WT matched groups. However, both TG and WT female groups exhibited higher GSH expression level when compared to matched male groups (Figure 1E). Considering that lower glutathione levels and depletion of antioxidant defense proteins are associated with increased ROS levels, we examined the expression level of NADPH oxidases (NOXs), which contribute mainly to ROS generation. Both proteins, NOX2 and NOX4, showed significantly increased expression in both TG males and females compared with the corresponding WT groups (Figures 1F,G). Notably, female sex showed significant NOX2 upregulation and NOX 4 downregulation when compared to matched male groups.

### Sex dependent differential HSPs expression and cardiomyocyte passive stiffness

**Figure 2:**
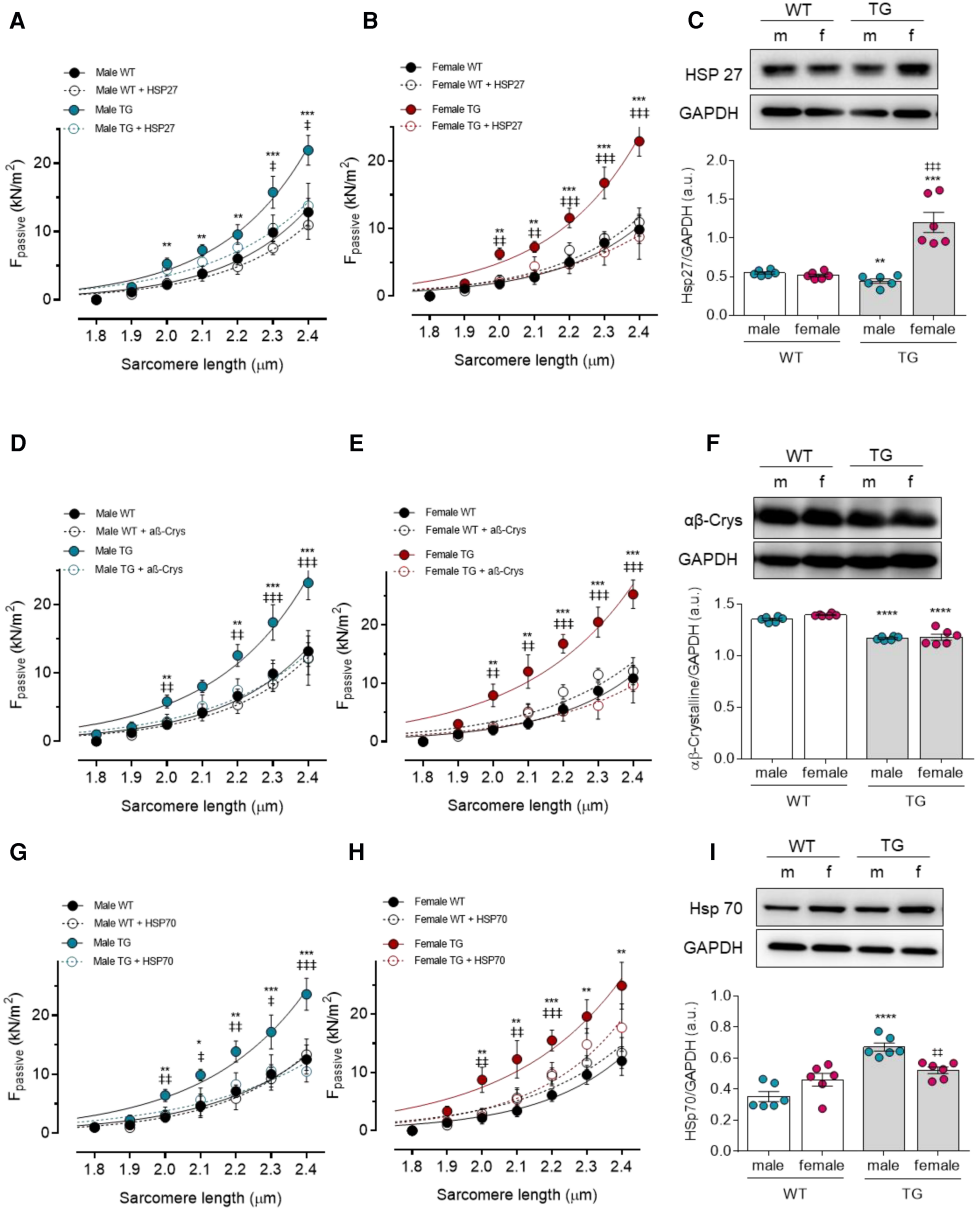
Effect of heat shock proteins (HSPs) on cardiomyocyte passive stiffness (F_passive_) in male and female mRen2 and WT rats. F_passive_ before (baseline) and after *in vitro* HSP27 administration at sarcomere length 1.8–2.4 *μ*m in (**A**) male and (**B**) female mRen2 and WT rats. (**C**) Expression level of HSP27 in male and female mRen2 and WT rats. F_passive_ before (baseline) and after *in vitro αβ*-Crystalline (*αβ*-Crys) administration in (**D**) male and (**E**) female mRen2 and WT rats. (**F**) Expression level of HSP27 in male and female mRen2 and WT rats. F_passive_ before (baseline) and after *in vitro* HSP70 administration in (**G**) male and (**H**) female mRen2 and WT rats. (**I**) Expression level of HSP70 in male and female mRen2 and WT rats. Data are shown as mean ± SEM; *n* = 6. Panel (**A–H**): *P < 0.05/**P < 0.01/***P < 0.001/****P < 0.001 female WT vs. female TG and male WT vs. male TG; ‡*P* < 0.05/‡‡*P* < 0.01/‡‡‡*P* < 0.001/‡‡‡‡*P* < 0.001 female TG vs. female TG + HSP27/ *αβ*-Crys / HSP70 and male TG vs. male TG + HSP27/ *αβ*-Crys / HSP70. Panel (**C, F, I**): *P < 0.05/**P < 0.01/***P < 0.001/****P < 0.001 female WT vs. female TG and male WT vs. male TG; ‡*P* < 0.05/‡‡*P* < 0.01/‡‡‡*P* < 0.001/‡‡‡‡*P* < 0.001 female WT vs. male WT and female TG vs. male TG 2-way ANOVA followed by Tukey's multiple comparisons test.

The molecular components of PQS, especially HSPs, can be targeted by oxidative modifications leading to deficient cytoprotective function and thereby cardiac proteotoxicity. Hence, we examined the effect of *in vitro* supplementation of HSP on titin-based myocardial stiffness as well as the expression level of various sHSP proteins (HSP27, *αβ*-crystallin, and HSP70). In general, treatment with HSP27, *αβ*-crystallin, and HSP70 reduced the significantly increased F_passive_ in both TG males and females compared to the untreated corresponding TG groups. WT cardiomyocytes from males and females showed no differences in F_passive_ with or without HSP treatment (Figures 2A,B,D,E,G,H).

As HSPs are present in cells under physiological conditions and are upregulated under stress conditions, we examined their expression in TG compared to WT animals. While HSP27 expression was significantly reduced in male TG rats compared to WT male rats, the HSP27 expression was significantly elevated in female TG rats compared to both WT female and TG male groups (Figure 2C). In contrast, a significant reduction in *αβ*-crystallin expression was found in male and female TG rats compared to the corresponding WT rats (Figure 2F). For HSP70, however, only a significant increase was found in male TG rats compared to both male WT and female TG groups (Figure 2I). These data suggest that the individual HSPs are differently regulated in a sex-dependent manner.

### Altered titin post translational modifications in male and female TG rats

**Figure 3:**
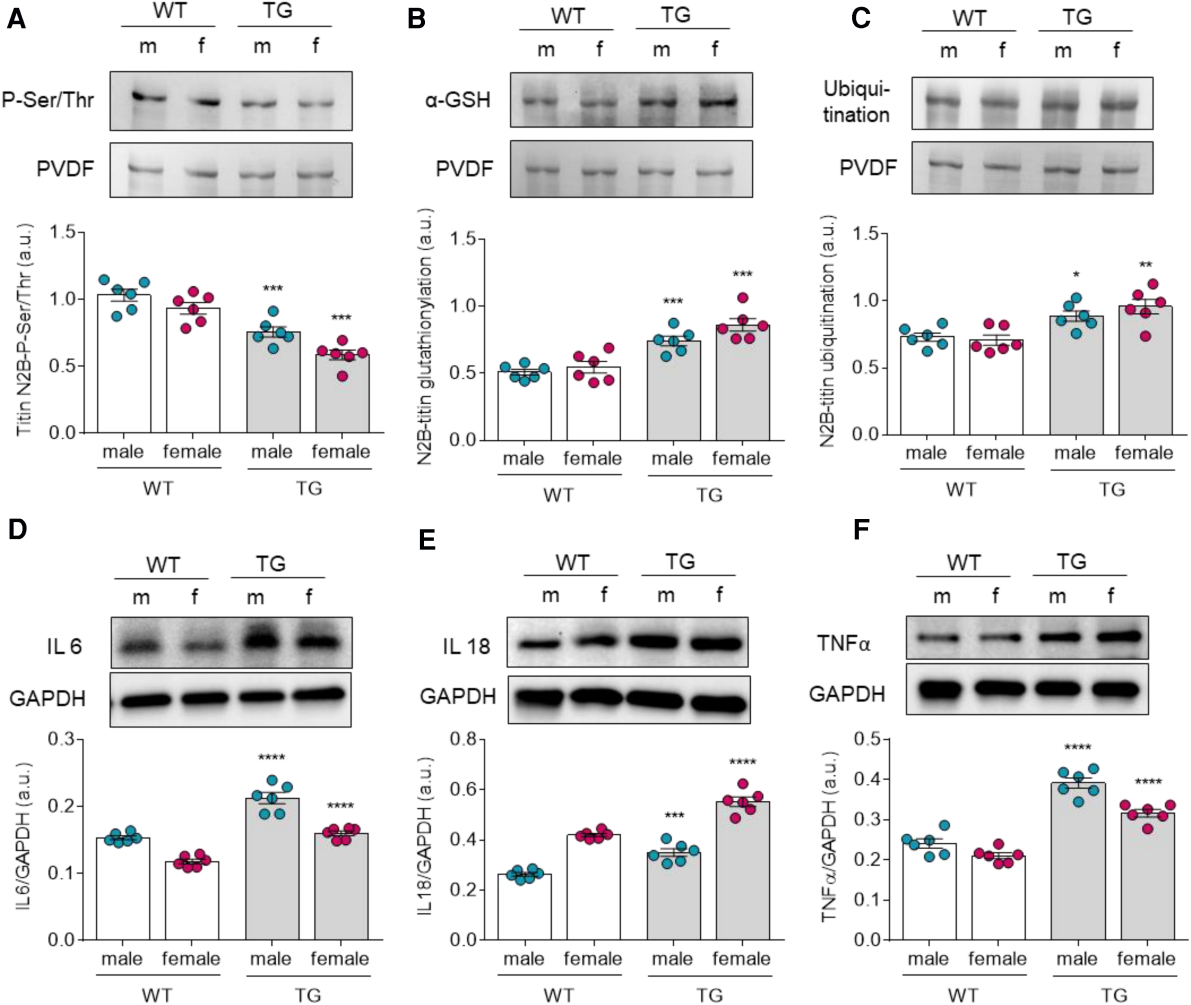
Altered titin post-translational modifications and inflammation markers in male and female mRen2 and WT rats. N2B-Titin (**A**) total phosphorylation, (**B**) total glutathionylation and (**C**) total ubiquitination. Expression levels of (**D**) interleukin 6 (IL6), (**E**) IL18 and tumor necrosis factor *α* (TNF*α*). Data are shown as mean ± SEM; *n* = 6. Panel (**A-F**): *P < 0.05/**P < 0.01/***P < 0.001/****P < 0.001 female WT vs. female TG and male WT vs. male TG; ‡*P* < 0.05/‡‡*P* < 0.01/‡‡‡*P* < 0.001/‡‡‡‡*P* < 0.001 female WT vs. male WT and female TG vs. male TG by 2-way ANOVA followed by Tukey's multiple comparisons test.

Other modulators of titin-based stiffness include posttranslational modifications that may vary by gender and pathology. Therefore, we investigated the phosphorylation status, oxidation state (S-glutathionylation), and ubiquitination of titin. While N2B phosphorylation of titin was significantly reduced in TG females and males compared with the corresponding WT groups (Figure 3A), N2B glutathionylation and N2B ubiquitination of titin were significantly increased in TG groups (Figures 3B,C). However, both TG and WT female groups showed no significant alterations in titin post translation modifications when compared to their matched male groups.

### Altered proinflammatory cytokine levels in male and female TG rats

Based on our findings of oxidative stress-related changes in titin, increased NADPH oxidase expression, and altered HSPs expressions in TG groups, it was also plausible to investigate inflammatory responses in all groups. Therefore, we examined the expression of proinflammatory cytokines such as IL-6, IL-18, and TNF*α*. All of which were significantly increased in diastolic dysfunction in TG compared to WT groups (Figures 3D,E,F). In addition, both male and female animals showed comparable tendencies towards higher cytokine levels in TG groups.

### Differential regulation of apoptotic pathways and proteases in male and female TG rats

**Figure 4:**
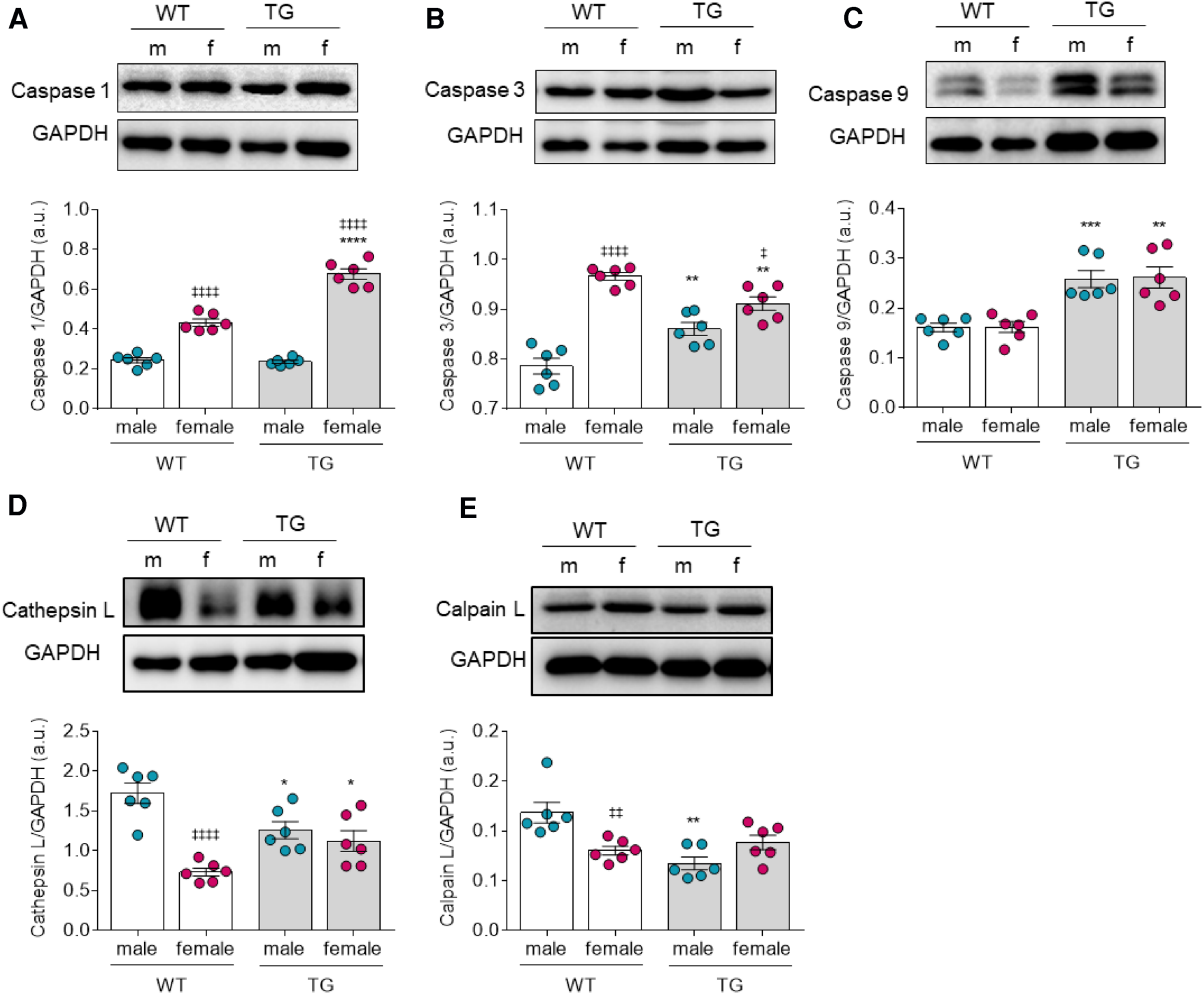
Apoptotic factors in male and female mRen2 and WT rats. Levels of (**A**) Caspase 1, (**B**) Caspase 3, (**C**) Caspase 9, (**D**) Cathepsin L and (**E**) Calpain L. (**F,G**) F_passive_ before (baseline) and after *in vitro* Caspase 3 inhibitor (Caspase3-i) administration at sarcomere length 1.8–2.4 *μ*m in (**F**) male and (**G**) female TG and WT rats. Data are shown as mean ± SEM; *n* = 6. Panel (**A–E**): *P < 0.05/**P < 0.01/***P < 0.001/****P < 0.001 female WT vs. female TG and male WT vs. male TG; ‡*P* < 0.05/‡‡*P* < 0.01/‡‡‡*P* < 0.001/‡‡‡‡*P* < 0.001 female WT vs. male WT and female TG vs. male TG; for Fpassive: *P < 0.05/**P < 0.01/***P < 0.001/****P < 0.001 female WT vs. female TG and male WT vs. male TG; ‡*P* < 0.05/‡‡*P* < 0.01/‡‡‡*P* < 0.001/‡‡‡‡*P* < 0.001 female TG vs. female TG + Caspase 3-inhibitor and male TG vs. male TG + Caspase 3-inhibitor by 2-way ANOVA followed by Tukey's multiple comparisons test.

Oxidative stress also plays a pivotal role in apoptosis. Therefore, we investigated the three functional caspase groups involved in (i) inflammatory cytokine processing such as caspase-1 (Figure 4A), (ii) apoptotic effector caspases-3 (Figure 4B), (iii) apoptotic initiator caspases-9 (Figure 4C), and additionally the proteases cathepsin L (Figure 4D) and calpain (Figure 4D). We found the expression level of caspase-1 to be significantly upregulated only in female TG rats compared to the matched control group but unchanged in male TG rats (Figure 4A). Of note, expression of caspase-3 was significantly increased in male TG rats, whereas it was significantly downregulated in female TG rats compared with the corresponding WT groups (Figure 4B). On the other hand, both caspase-1 and caspase-3 were significantly upregulated in TG and WT female groups when compared to the matched male groups (Figures 4A,B). The expression of caspase-9 was significantly upregulated in both male and female in TG rats (Figure 4C). Both proteases, cathepsin and calpain (Figures 4D,E), showed a significant reduction in male TG rats compared to WT rats. In contrast, female TG rats showed only a slight increase in expression of both proteases compared to female WT rats, however, the increase was only statistically significant for cathepsin L. Furthermore, cathepsin L and calpain L were downregulated in female WT compared to male WT group (Figure 4D).

### Altered autophagy response in male and female TG rats

**Figure 5:**
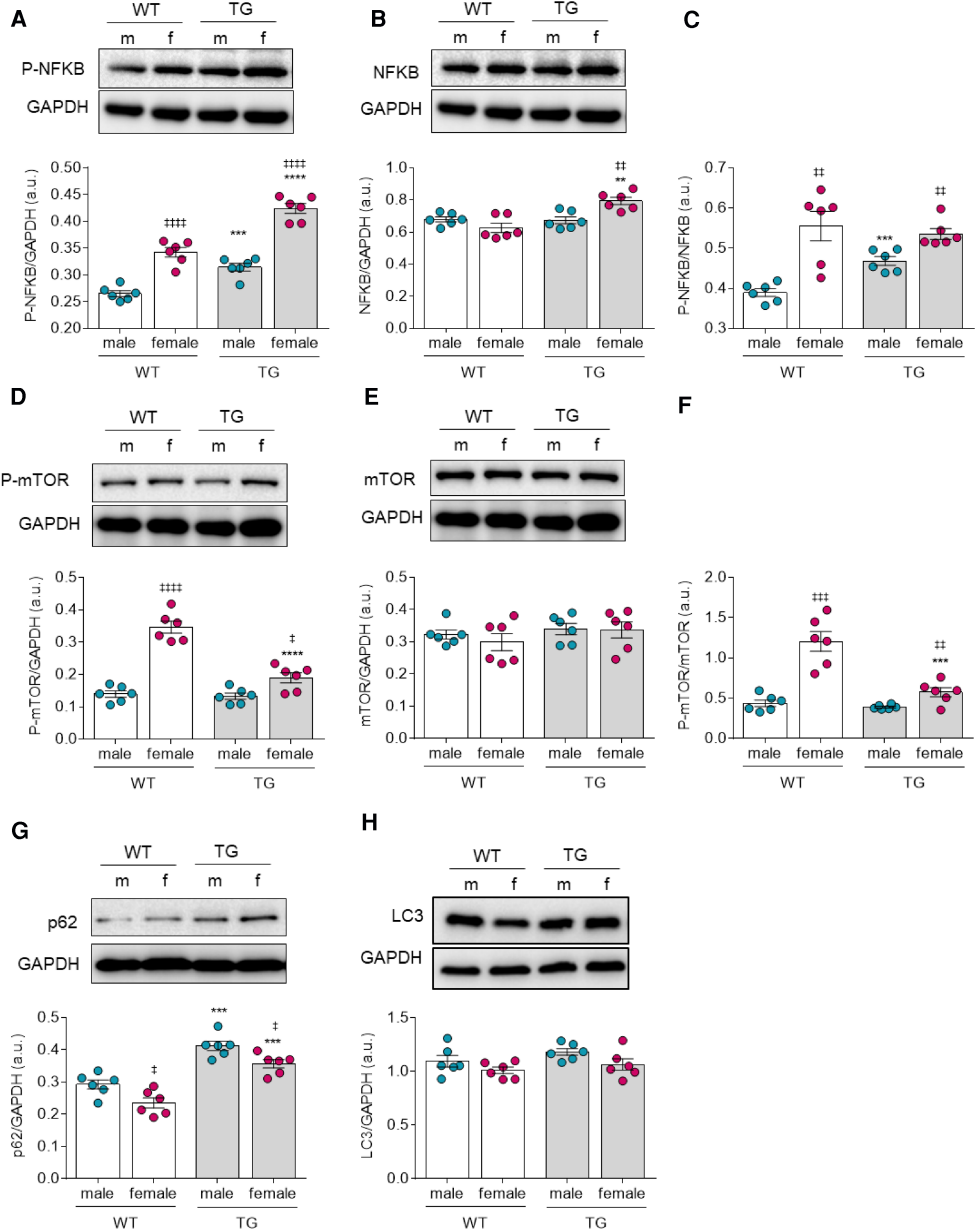
Markers of autophagy in male and female mRen2 and WT rats. (**A**) Phosphorylation, (**B**) expression and (**C**) ratio of phosphorylated over total Nuclear factor kappa-light-chain-enhancer of activated B cells (NF-*κ*B). (**D**) Phosphorylation, (**E**) expression and (**F**) phosphorylated/total mammalian target of rapamycin (mTOR)-ratio. Expression level of (**G**) Sequestosome 1 (p62) and (**H**) autophagy marker light chain 3 (LC3). Data are shown as mean ± SEM; *n* = 6. Panel (**A–F**): *P < 0.05/**P < 0.01/***P < 0.001/****P < 0.001 female WT vs. female TG and male WT vs. male TG; ‡*P* < 0.05/‡‡*P* < 0.01/‡‡‡*P* < 0.001/‡‡‡‡*P* < 0.001 female WT vs. male WT and female TG vs. male TG by 2-way ANOVA followed by Tukey's multiple comparisons test.

Furthermore, we examined the phosphorylation status of NF-*κ*B, which is involved in stress responses and plays a central role in mediating immune and inflammatory responses along with regulating cell proliferation, apoptosis, and autophagy. We found that NF-*κ*B phosphorylation level was significantly increased in both TG groups compared to their matched WT groups (Figure 5A). However, the total amount of NF-*κ*B in female TG rats also showed a significant increase compared with the corresponding female control group (Figure 5B). Therefore, the ratio of NF-*κ*B phosphorylation over total NF-*κ*B was only significantly elevated in TG male rats compared with matched control rats. Of note, both TG and WT female groups exhibited greater NF-*κ*B phosphorylation over total NF-*κ*B when compared to the matched male groups.

In addition, mTOR (mammalian target of rapamycin) is also a regulator of various signaling pathways such as autophagy, apoptosis, and cell growth, hence we further investigated changes in its phosphorylation level. mTOR phosphorylation was significantly downregulated only in female TG rats and remained unchanged in male TG rats compared to WT male rats (Figure 5D). The expression level of total protein among all groups was unchanged. Consequently, the ratio of phosphorylated to total protein in female TG rats was significantly decreased but remained unchanged in male rats compared to control groups (Figure 5F). However, when compared to their matched male groups, both TG and WT female groups exhibited higher phosphorylated to total mTOR ratio (Figure 5F). In addition, we examined downstream effectors that play an important role in cellular autophagy, such as the ubiquitin-binding protein p62 (sequestosome 1), which is an autophagosome cargo protein, together with the autophagy marker light chain 3 (LC3). The expression level of p62 was significantly elevated in both male and female TG groups compared with the control groups. However, both TG and WT male groups showed greater elevation in p62 level compared to matched female groups (Figure 5G). LC3 expression level remained unchanged all groups (Figure 5H).

## Discussion

A wide variety of comorbid conditions and risk factors correlate significantly with HFpEF phenotype. Longstanding arterial hypertension, among others like type 2 diabetes, is well known as a potential cofounder of myocardial structural and functional changes and the subsequent diastolic dysfunction (27). Although, our understanding of diastolic dysfunction as a fundamental contributor in HFpEF pathology has been substantially advanced, the treatment options remained limited due to heterogeneity in the mechanisms arising from the comorbid conditions and risk factors (28). Female sex is differentially recognised in the context of disease prevalence, functional parameters, and treatment outcomes (29, 30). However, a deep understanding of the sex-dependent alterations in molecular mechanisms that drive the diverse mechanical and functional abnormalities is still lacking. In the current study we analysed sex-specific differences of key molecular mechanisms involved in cardiac remodelling and diastolic dysfunction.

In both sexes, we demonstrated a significant elevation in F_passive_ of TG cardiomyocytes, which is a major determinant of diastolic dysfunction. In agreement with previous research highlighting the association between oxidative stress and increased myocardial stiffness, we detected high levels of oxidative stress in TG rats with prominent effects in female rats. *In vitro* supplementation with sHSPs reversed the elevated F_passive_ indicating restoration of their cytoprotective function. Furthermore, TG rats exhibited high levels of proinflammatory cytokines in addition to significant alterations in apoptotic and autophagy pathways in both sexes.

### Elevated titin-based myocardial stiffness due to oxidative stress in TG animals

Hypertension is a well-characterized risk factor for diastolic dysfunction. The chronic systemic pressure overload correlates with maladaptive cardiac remodelling processes including LV hypertrophy and fibrosis, thereby leading to reduced myocardial relaxation and diastolic compliance (27). The increased myocardial stiffness is a primary feature of diastolic dysfunction (31). Consistently, we found in both sexes a significant elevation of F_passive_ in TG cardiomyocyte, indicative of diminished diastolic compliance. Since oxidative modifications of myofilament proteins and kinases are known to modulate myocardial stiffness (32), the reduction in cardiomyocyte passive stiffness upon GSH supplementation and in TG cardiomyocytes suggests a subtle role of oxidative stress in the modulation of myocardial stiffness, perhaps via altering titin post-translational modifications in TG cardiomyocytes such as phosphorylation and oxidations. This is in line with the significant decrease in GSH content in cardiomyocytes of TG rats compared to matched control groups and the upregulation of NADPH oxidases (NOX) the crucial mediators of ROS generation and inflammatory responses (33). Indeed, in TG animals, angiotensin-2 (ANG II) was found to stimulate NADPH oxidase-dependent-ROS production mainly through the activation of the MAPK signalling pathways (34). Of note, the NOX2 expression level showed a pronounced elevation in female TG cardiomyocytes compared to all groups. In agreement with this result, female sex has been suggested to exhibit higher tendencies towards increased levels of oxidative stress and inflammation (14). Both mechanisms mediate diastolic dysfunction via endothelial, ECM and cardiomyocyte dysfunction (16).

### Effects of oxidative stress on titin and PQS components in TG animals

When under stress conditions ROS generation exceeds the antioxidative capacity, a direct oxidative modification of the proteins can cause functional and/or structural impairments that lead to protein misfolding, aggregation, and increased myocardial stiffness. Small HSPs (sHSPs) are fundamental components of the PQS serving as a first line of defence against protein misfolding (7). In agreement with previous studies reporting the upregulation of sHSPs upon various stress conditions (35), our data showed distinct regulation of HSP27 and HSP70 in female compared to male TG rats. This distinct regulation suggests the existence of sex-specific regulation of the PQS pathways. Despite the upregulation of endogenous sHSP in TG animals, cardiomyocyte passive stiffness remained elevated and was reversed to control levels only after *in vitro* supplementation of sHSPs in both male and female TG cardiomyocytes. Such observation can be explained by direct and/or indirect effect of oxidative modifications perhaps of HSPs, translocation of HSP away from sarcomeres or HSPs proteins malfunction (15–20). Previously, we reported that sHSPs can be targeted by ROS leading to a reduction of their cytoprotective function and hence protein aggregation (19, 20). In human hypertrophic cardiomyopathy (HCM), we detected oxidative-stress induced impairments in PQS as anticipated from the S-glutathionylation of HSP 27 and *αβ*-crystallin (20). Furthermore, we and other reported a oxidative stress-induced translocation of HSP27 and *αβ*-crystallin away from the Z-disk and A-band in HCM (19, 36). Conversely, sHSPs supplementation reduced the elevated F_passive_ in HCM cardiomyocyte (19, 20), further confirming the direct effect of oxidative modifications on PQS.

Through its mechano-sensing properties, titin represents the main determinant of the cardiomyocyte passive stiffness. Previous research by us and others demonstrated post-translational modifications of titin such as phosphorylation, ubiquitination, and oxidation within cardiomyocyte regulate or modify the myocardial stiffness. Oxidative modifications of titin have been linked to impaired diastolic stiffness in HF patients (15, 19, 23). In the current study, we detected high levels of titin S-glutathionylation and ubiquitination in both male and female TG rats compared to matched control groups. Titin can be oxidized in different ways, either forming disulphide bridges, by S-glutathionylation, or S-nitrosylation leading either to an increase or reduction of the acrdiomyocyte stiffness (37, 38). An important mechanism that regulates titin elasticity under physiological and oxidative conditions is the mechanical unfolding of I-band Ig-domains (39). Through increased mechanical strain on the sarcomeres, Ig domains unfold, thereby exposing the cryptic cysteines for redox modifications and resulting in the formation of disulphide bridges or S-glutathionylation (38). S-glutathionylation of the cryptic cysteines at the Ig domains prevents their refolding, decreases their mechanical stability, and reduces the passive tension (38). On the other hand, disulphide bonding in the N2B-us domain reduces its extensibility resulting in elevated cardiomyocyte passive tension (40). Upon stress, molecular chaperons such as HSP27 and *αβ*-crystallin, translocate to sarcomeres and bind at specific I-band regions of titin protecting thereby the unfolded Ig domains from aggregation and the consequent myocardial stiffening (36). However, direct oxidative modifications of these chaperons and/or their binding partners prevents the correction of misfolded proteins and hinders their clearance by the proteasome machinery (19, 20). The accumulation of protein aggregates may result in elevated myocyte stiffness, aggravated oxidative stress, and augmented cell death pathways (35, 41). These mechanisms might explain the elevation oftitin ubiquitination and cardiomyocyte stiffness in TG animals despite the upregulation of sHSPs expression. Notably, differential upregulation of sHSPs in male vs. female TG animals was observed, suggesting sex-specific regulation of sHSP.

Oxidative stress may also modulate cardiomyocyte function via indirect effects on several signalling pathways involved in posttranslational modifications of myofilament proteins (17). Oxidation of kinases and/or their downstream targets results in dysregulated phosphorylation of several proteins (17). Among which, the deranged phosphorylation status of titin due to oxidative-stress induced impairments in several kinases that phosphorylate titin spring elements (26, 42). In failing human hearts, PKA and PKG dependent hypo-phosphorylation of titin is associated with increased myocardial stiffness (25), which could be reversed upon kinase supplementation and anti-oxidant treatment (17, 23). In TG animals we detected in addition to titin S-glutathionylation, a significant reduction in total titin phosphorylation, suggesting potential alterations in kinase/phosphatase-titin interactions, which might result in dysregulated phosphorylation/dephosphorylation processes and hence altered myocyte stiffness. Thus, titin oxidation could play a major role in modulating passive stiffness, similar to or togetherwith the effects of titin phosphorylation.

### Elevated pro-inflammatory cytokines in TG animals

In HFpEF patients, and upon various comorbid conditions, the systemic inflammatory state and microvascular inflammation have been linked to myocardial dysfunction (43). Pro-inflammatory cytokines induce ROS generation thereby exacerbating oxidative stress. In addition, pro-inflammatory signals contribute to myocardial fibrosis and stiffness, mainly via macrophage stimulation and/or collagen formation by fibroblast activation (12, 13). In TG animals, pro-oxidant agonists, such as Ang II and tumour necrosis factor- *α* (TNF-α) are known to induce the expression of pro-inflammatory molecules. Indeed, both IL-6 and IL-18 showed significant elevation in both sexes. However, distinct elevation patterns were found in male vs. female TG animals confirming the sex-specific differences in pro-inflammatory responses to hypertensive conditions.

### Altered apoptotic and autophagy pathways in TG animals

The activation of apoptotic cascades occurs in response to the accumulation of oxidized, aberrant proteins upon PQS dysfunction (44). The diminished ability of sHSPs to inhibit apoptosis may also contribute to the increase in apoptotic events (45, 46). Furthermore, chronic mechanical overload associates with increased ROS generation, hypertrophic remodelling, and apoptosis. Therefore, we checked the expression level of apoptotic markers and found caspase-3 and caspase-9 to be significantly upregulated in both male and female TG animals compared to control groups. In addition, the expression levels of proteases such as cathepsin and calpain were differentially regulated in male and female TG animals compared to matched control groups. These results suggest a contribution of dysregulated apoptotic and proteolytic pathways to diastolic dysfunction. Interestingly, caspase-1 showed significant upregulation in female but not in male TG animals compared to matched control groups. Consistently, previous studies reported more frequent apoptotic event in female compared to male sex under both physiological and pathological conditions (47, 48). Caspase-1 is activated in inflammasomes and was shown to trigger both programmed necrosis (pyroptosis) and apoptotic pathways (49). In addition, caspase-1 activation promotes IL-18 release and NF-κB activation (50). Both of which showed higher upregulation/activation tendencies in female compared to male TG animals. These observations can be attributed to the sex-specific differences in immune responses, as the susceptibility to inflammatory events and autoimmune diseases is generally higher in females (14).

It is evident that dysregulated autophagy plays a pivotal role in PQS dysfunction. Down or upregulated, autophagic responses were shown to be involved in pathological cardiac remodelling upon various stress conditions (51). In the current study, female TG animals exhibited a significant decrease in mTOR phosphorylation level, compared to matched control group. However, both TG sexes showed significant upregulation in sequestosome-1, also known as ubiquitin-binding protein p62, which is an autophagosome cargo protein, suggesting the contribution of alteredNFkB and mTOR in PQS dysfunction and diastolic impairments in TG animals (52).

### Molecular mechanisms underlying sex-dependent differences in diastolic dysfunction

Clinical data from HFpEF cohorts demonstrate the existence of sex-dependent differences in terms of disease progression, prognosis, and therapy outcomes (53). In addition, sex-dependent comorbid conditions have been suggested to influence the diverse HF phenotypes (53). Although the mechanisms underlying these variations have yet to be explained, it is evident that multiple factors contribute to the sex-specific differences in disease development such as sex-hormones, immune response, risk factors, and the Y chromosome (54). Testosterone was suggested to be the main driving force for hypertension in men (48). Moreover, postmenopausal women exhibit higher prevalence of LV diastolic dysfunction than men suggesting a cardio-protective role of female sex-hormones, however inconsistent findings were reported about the impact of hormone replacement therapy on the elevated blood pressure in women (55, 56). In both sexes, activation of the Renin-Angiotensin-Aldosteron-System (RAAS) is associated with hypertension, cardiac hypertrophy, cardiac fibrosis, and impaired cardiomyocyte relaxation. Mounting amount of evidence demonstrate the regulatory role of estrogen on RAAS activity (57), NO bioavailability (58), and myocardial substrate metabolism (59). However, it is evident that hypertension increases the risk of HF by 3 times in women compared to only twice in mRen2 (60). Furthermore, women frequently develop diastolic HF, and men more often systolic HF (61, 62). A plausible explanation for such tendencies despite the protective role of female hormones is the complex interaction between oxidative stress, inflammation, hormones, and sex-specific gene regulation (54).

A classic example of such complex interplay is LV hypertrophy. LV hypertrophy, a major causative factor in reduced diastolic compliance, is mediated by AKT signalling, which is known to have higher activity in women compared to men hearts (63). Other estrogen-sensitive pathways include mTOR, GSK3ß, MAPK-ERK1/2. All of which can be dysregulated in the presence of inflammation and oxidative stress impairing thereby PQS activity (20). Therefore, the degree of redox-imbalance, inflammation, and PQS dysfunction potentially contribute to the observed sex differences in terms of diastolic dysfunction.

## Conclusion

In summary, the data presented in the current study provide evidence ofdiastolic dysfunction in TG animals, which is associated with impaired cardiomyocyte function and impaired vasodilator responses through increased systemic inflammation and oxidative stress. TG animals also exhibited PQS impairment as anticipated from sHSPs malfunction and the alterations of many signal transduction pathways that are involved in autophagy and apoptosis. Our work provided further evidence of sex-specific mechanisms in the development of diastolic dysfunction in HF animals. Therefore, future research is needed to unravel the sex-dependent mechanisms contributing to HF pathology in order to design sex-specific and thus more effective therapies for female or male HF-patients.

## Data Availability

The raw data supporting the conclusions of this article will be made available by the authors, without undue reservation.
